# Global disparities in public health guidance for the use of COVID-19 vaccines in pregnancy

**DOI:** 10.1136/bmjgh-2021-007730

**Published:** 2022-02-24

**Authors:** Eleonor Zavala, Carleigh B Krubiner, Elana F Jaffe, Andrew Nicklin, Rachel Gur-Arie, Chizoba Wonodi, Ruth R Faden, Ruth A Karron

**Affiliations:** 1 Department of International Health, Bloomberg School of Public Health, Johns Hopkins University, Baltimore, Maryland, USA; 2 Center for Global Development, Washington, District of Columbia, USA; 3 Berman Institute of Bioethics, Johns Hopkins University, Baltimore, Maryland, USA; 4 School of Medicine, University of North Carolina at Chapel Hill, Chapel Hill, North Carolina, USA; 5 Centers for Civic Impact, Krieger School of Arts & Sciences, Johns Hopkins University, Baltimore, Maryland, USA; 6 Department of International Health, International Vaccine Access Center at the Bloomberg School of Public Health, Baltimore, Maryland, USA

**Keywords:** vaccines, COVID-19, maternal health, health policy

## Abstract

**Introduction:**

Gaps in information about the safety and efficacy of COVID-19 vaccines in pregnancy have led to substantial global variation in public health guidance regarding the use of COVID-19 vaccines in pregnancy over the course of the pandemic.

**Methods:**

We conducted systematic screenings of public health authorities’ websites across 224 countries and territories every 3 weeks to track the development of policies on COVID-19 vaccine use in pregnancy. Policies were categorised using a 1–5 permissiveness scale, with 1 indicating policies that recommended use, and 5 indicating policies that recommended against use.

**Results:**

As of 30 September 2021, 176 countries/territories had issued explicit guidance on COVID-19 vaccine use in pregnancy, with 38% recommending use, 28% permitting use, 15% permitting use with qualifications, 2% not recommending but with exceptions, and 17% not recommending use whatsoever. This represented a significant shift from May 2021, when only 6% of countries/territories with such policies recommended the use of COVID-19 vaccines in pregnancy (p<0.001). However, no policy positions could be found for 21% of all countries and territories, the vast majority being low and middle income. Policy positions also varied widely by vaccine product, with Pfizer/BioNTech and Moderna vaccines being most commonly recommended or permitted.

**Conclusion:**

Our study highlights the evolution of policies regarding COVID-19 vaccine use in pregnancy over a 5-month period in 2021, the role of pregnancy-specific data in shaping these policies and how inequities in access for pregnant people persist, both within countries and globally.

**Key questions**

**What is already known?**

- A number of COVID-19 vaccines have received WHO authorisation and national regulatory authority authorisation or licensure.
- Compared with other adults of similar age, pregnant people are at increased risk of severe COVID-19 and death; however, national policies for use of COVID-19 vaccines during pregnancy vary widely by geography and over time, and to our knowledge, have not been systematically assessed.

**What are the new findings?**

- The number of pregnancy-specific policies for the use of COVID-19 vaccines across 224 countries and territories increased substantially, from 133 in May to 176 in September; and the proportion recommending or permitting use of at least one COVID-19 vaccine in pregnancy also increased, from 32% in May to 66% in September.
- However, 25% of low/middle-income countries (LMICs) recommended against the use of COVID-19 vaccines in pregnant people, with several countries citing the absence of pregnancy-specific clinical trial data and developmental and reproductive toxicology data in their decisions to limit access to COVID-19 vaccines for pregnant people.
- Permissiveness varied by vaccine platform. Recommendations for Oxford-AstraZeneca vaccine were substantially less permissive than recommendations for mRNA vaccines, even in countries where Oxford-AstraZeneca is the only vaccine available and high rates of community transmission exist, and thus where the WHO supports its administration in pregnancy.

**Key questions**

**What do the new findings imply?**

- While some progress has been made in adopting policies to promote access to COVID-19 vaccines for pregnant people, the persistence of restrictive policies, particularly in LMICs, is a threat to health and health equity for these high-risk individuals.
- In the near term, policies should be revised and developed to promote access to COVID-19 vaccines for pregnant people, including greater global access to vaccines for which post-authorisation safety evidence in pregnancy is available.
- Going forward, pregnant people should be included in clinical vaccine trials for emerging pathogens.

## Introduction

Global deployment of COVID-19 vaccines is essential for saving lives in the SARS-CoV-2 pandemic.^1–4^ A majority of countries have developed policies to guide COVID-19 vaccine use. While most countries have prioritised vaccination of healthcare workers, the elderly and other populations at increased risk of infection or severe disease, recommendations related to COVID-19 vaccine use in pregnancy vary widely. Pregnancy has historically been an exclusion criterion for vaccine research and deployment during infectious disease outbreaks and epidemics.^5^ Despite recent work that has highlighted the need for inclusion of pregnant people in vaccine trials for emerging pathogens,^5 6^ they were excluded from phase III COVID-19 vaccine trials. Consequently, data regarding the safety and efficacy of COVID-19 vaccines in pregnancy have been extremely limited, complicating country decision-making.

At the same time, evidence continues to mount that pregnant people infected with SARS-CoV-2 are at increased risk of severe disease, hospitalisation and death compared with non-pregnant people.^7–11^ They are also at higher risk of pre-eclampsia, preterm birth and of having babies that require intensive care, compared with pregnant people not infected with SARS-CoV-2.^12–14^ More recently, infection with SARS-CoV-2 variants, including Alpha and Delta, has been associated with greater risks of adverse maternal and fetal outcomes than reported with wild-type SARS-CoV-2.^15^ With respect to vaccine safety, data from developmental and reproductive toxicology (DART) studies have been reassuring where available,^16–18^ as are observational data derived from use of authorised mRNA vaccines in pregnant people, primarily collected in high-income settings.^19–25^ Notably, real-world safety data for other COVID-19 vaccine platforms and for pregnant people in low/middle-income country (LMIC) settings are lacking.

Despite these information gaps, numerous countries and the WHO now include pregnant people alongside those at elevated risk of severe COVID-19 in vaccine prioritisation plans.^26 27^ To understand the global variance in vaccine policy related to COVID-19 vaccine use in pregnancy, we systematically searched for documents posted by public health authorities (PHAs) and ministries of health (MOHs) from 224 countries and territories and reviewed their positions on COVID-19 vaccine use in pregnancy. Understanding variations in recommendations for use of COVID-19 vaccines in pregnancy, as well as the stated rationale for these policies, is valuable for policymakers, clinicians and pregnant people. To make these data broadly available, we developed a website (https://www.comitglobal.org) in which these policies are catalogued and regularly updated. Here, we offer a global snapshot of national policies as of 30 September 2021 and describe their evolution between May and September 2021.

## Methods

### Search strategy and selection criteria

We compiled a dataset of national PHA web pages that contained information about COVID-19 vaccines. These were identified through search engines using keywords related to (1) authoritative bodies (MOH; centre for disease control); (2) COVID-19 vaccines (national vaccine plan; priority groups; vaccine rollout); and (3) country/territory name. The list of countries and territories was based on the COVID-19 Global Education Recovery Tracker (https://www.covideducationrecovery.global/), a Johns Hopkins University/World Bank/UNICEF partnership. On 3 May 2021, we began systematically screening PHA websites for all 224 countries and territories in 3-week intervals. We searched web pages for eligible documents, including vaccine guidance statements, fact sheets, frequently asked questions (FAQs), press releases, official social media posts, screening checklists and government web pages. Media articles and unofficial social media accounts were not eligible. If posted in a language other than English, documents were translated by data collectors fluent in the language or via Google Translate (https://translate.google.com/). In the infrequent instances where a website for a country authority was inactive or inaccessible from the USA (where the authors are located), colleagues in those countries were contacted to request documentation, if available.

### Data extraction

For each document, we extracted country and authority name, type of document, date published or last updated, vaccine products if specified, the pregnancy guidance free text, any qualifications or subgroup specifications for vaccine administration among pregnant individuals (eg, high-risk groups), justifications for the position taken (eg, lack of data) and vaccine product preference language. When documents made no mention of vaccine administration in pregnancy, we collected the authority, country and document details to flag guidance concerning eligibility without specifications for pregnant populations. All entries were time stamped.

### Coding policy positions

After an initial review of 33 national policies, we developed five categories to capture variation in national recommendations for use of COVID-19 vaccines (table 1). Category 1 includes policies that *recommend* use for all or some pregnant people (eg, those at high risk). Category 2 includes policies that *permit* use in pregnancy without restrictions. Some category 2 countries identify subgroups of pregnant people for whom the vaccine is particularly likely to have a positive risk–benefit ratio; however, they do not place restrictions on access in pregnancy. Category 3 includes policies that *permit* use but only for specific groups of pregnant people, most typically, those at high risk due to underlying conditions or background risk of infection. Category 3 also includes policies that place other qualifiers on access, such as requirements that pregnant people seek a provider consultation or prescription as conditions for vaccination. Category 4 includes policies that offer a general statement that the COVID-19 vaccine is *not recommended* in pregnancy, but specify instances in which exceptions can be made. Category 5 captures policies in which use is *not recommended* or *contraindicated* in pregnancy, with no exceptions provided. Policies were coded as ‘no position’ if no language regarding pregnancy was found or if the position was not clearly established, for example, ‘if pregnant, talk to your doctor’.

**Table 1:** Categorisation of policies for COVID-19 vaccine use in pregnancy

Category		Description	Example
1	Recommended for some or all	Pregnant people should receive COVID-19 vaccines; it is advised that pregnant people receive COVID-19 vaccines.	Mexico 11 May 2021 *Based on the findings of the preclinical phases of research, in animal models… and of the monitoring platforms of pregnant and vaccinated women against COVID-19…, it is considered that the benefits of vaccination for pregnant women outweigh the possible risks - real or theoretical - of vaccination in this population group, so people who are pregnant will be vaccinated against the SARS-CoV-2 virus*.^39^
2	Permitted	Pregnant people can choose to receive COVID-19 vaccines.	India 02 July 2021 *MoHFW has approved vaccination of pregnant women against COVID-19 with the condition that the pregnant women may be informed about the risks of exposure to COVID-19 infection along with the risks and benefits associated with the COVID-19 vaccines available in the country. Based on the information provided, a pregnant woman will have the choice to take the vaccination*.^40^
3	Permitted with qualifications	Pregnant people can choose to receive COVID-19 vaccines *only if certain conditions are met* (eg, individual pregnant people have high risk of exposure or severe disease, prescription or provider consultation is required).	South Africa 29 April 2021 *In the interim, pregnant women should receive Ad26.COV2.S only if the benefit of vaccination to the pregnant woman outweighs the potential vaccine risks, such as if the woman is a health worker at high risk of exposure or has comorbidities that place them in a high-risk group for severe COVID-19*.^41^
4	Not recommended but with exceptions	It is not recommended that pregnant people receive the COVID-19 vaccine *unless certain conditions are met* (eg, individual pregnant people have high risk of exposure or severe disease, prescription or provider consultation is required).	Guatemala 10 March 2021 *Pregnancy can increase the risk of developing a severe case of COVID-19 disease, though at this time COVID-19 vaccines are not currently recommended for all pregnant women, unless the risk of exposure to the virus is high (for example, if working in the health sector*).^42^
5	Not recommended	Pregnant people are not able to receive COVID-19 vaccines.	Angola 15 January 2021 *It should be noted that vaccination is not recommended for pregnant women*.^43^
6	No position found	Includes policies/countries where (1) no policies regarding vaccine rollout could be found; (2) policies and plans regarding eligibility could be found, but no positions regarding pregnancy specifically; or (3) no position was clearly established.	Eswatini 31 March 2021 *What you should tell your vaccination provider before vaccination:* *Tell your vaccination provider about all of your medical conditions, including if you are… pregnant or plan to become pregnant*.^44^

MoHFW, Ministry of Health and Family Welfare.

Three data collectors were trained by senior investigators. Collectors flagged policies where positions were unclear and these were double coded. When disagreements occurred, three team members, including two senior investigators, reviewed the document and together determined the most appropriate code.

### Country-level coding

Countries could have multiple policy positions for several reasons. Every document that mentioned vaccine administration in pregnancy was coded, so an authority’s guidance statement and their vaccine FAQ were entered and coded separately. Some countries might have two or more authorities issuing guidance, for example, an MOH and a national immunisation technical advisory group. When policy documents differed in coding, for example, a guidance statement was coded as a ‘3’, permitted with qualifications, but the FAQ published at the same time was coded as a ‘4’, not recommended but with exceptions, the overall country code would be categorised as the more permissive of the two.

Many countries took different positions for different vaccine products, so product-specific guidance resulted in multiple policy positions. To create global snapshots, countries were assigned a country-level code based on the most permissive policy across vaccine products. For example, if an authority issued guidance permitting use of Pfizer-BioNTech vaccine in pregnancy without restrictions but prohibited use of Oxford/AstraZeneca vaccine, the product-specific codes would be ‘2’ and ‘5’, respectively, and the country-level code would be categorised as a ‘2’.

### Regular screenings

The first systematic screening began on 3 May and was completed on 21 May. Subsequent screenings were completed in 3-week cycles; one-third of all countries/territories were screened and updated each week. Policies were entered into the database and country-level codes were automatically updated.

### Statistical analysis

Fisher’s exact test was used to compare differences in proportions.

### Patient and public involvement

Patients and the public were not involved in the design and conduct of this review of existing national policies. However, data that informed this review are available to the public through our website (wwwcomitglobalorg).

## Results

### Global variations in policy over time

**Figure 1:**
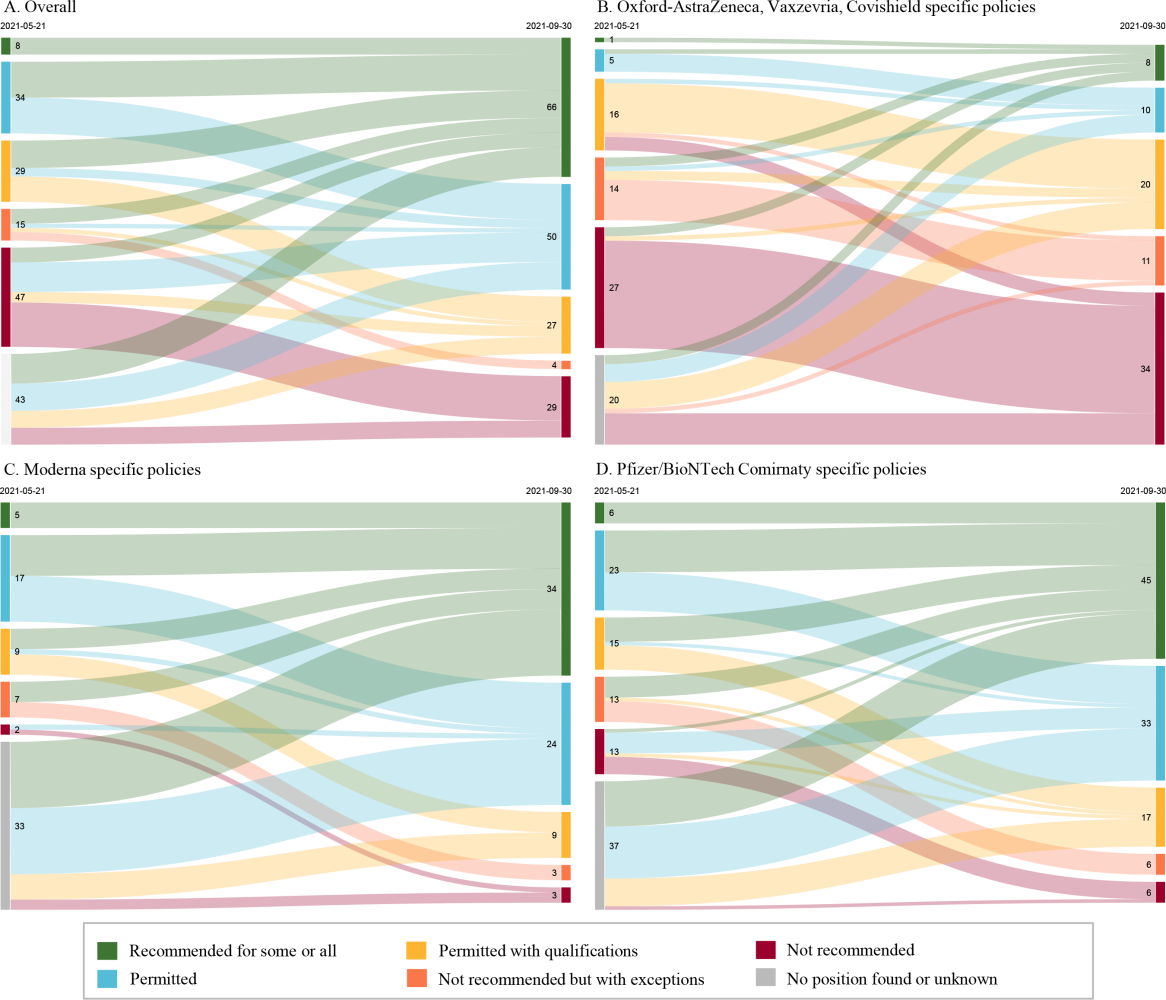
Trends in policies for use of COVID-19 vaccines in pregnancy, May–September 2021. For each panel, May 2021 is shown on the left and September 2021 is shown on the right. (A) Counts of countries/territories categorised by their most permissive COVID-19 vaccine policies for use in pregnancy across any vaccine product. Countries/territories where no position on COVID-19 vaccine use in pregnancy could be found were omitted from the diagram. (B) Counts of product-specific policies for the Oxford-AstraZeneca, Vaxzevria and Covishield vaccines. (C) Counts of product-specific policies for the Moderna COVID-19 vaccine. (D) Counts of product-specific policies for the Pfizer/BioNTech Comirnaty vaccine.

As of 21 May, we found documents related to COVID-19 vaccine use and eligibility for 158 countries/territories, some dating back to December 2020. Eight recommended use of a COVID-19 vaccine for some or all pregnant people, 34 permitted use for pregnant people, 29 permitted use with qualifications, 15 did not recommend use in pregnancy but with exceptions, 47 countries/territories did not recommend use in pregnancy (figure 1A). For 25 countries/territories, policy documents on COVID-19 vaccine eligibility were available but no position on use in pregnancy was included. Vaccine eligibility guidance was missing for 66 countries and territories.

Between May and September, we observed an increase in the total number of recommendations and in the permissiveness of pregnancy-specific policies (figure 1A). In May, 133 countries had a pregnancy-specific policy (categories 1–5), whereas in September, 176 countries had such policies. Moreover, the proportion recommending use had increased significantly (category 1; 8 of 133, 6.0% vs 66 of 176, 37.5%; p<0.001), as had the proportion either recommending or permitting use (categories 1 and 2, 42 of 133, 31.6% vs 116 of 176, 65.9%; p<0.001). Gaps in policy remained, however, as 48 countries/territories continued to have missing information.

### Global distribution of COVID-19 vaccine policies in pregnancy

**Figure 2:**
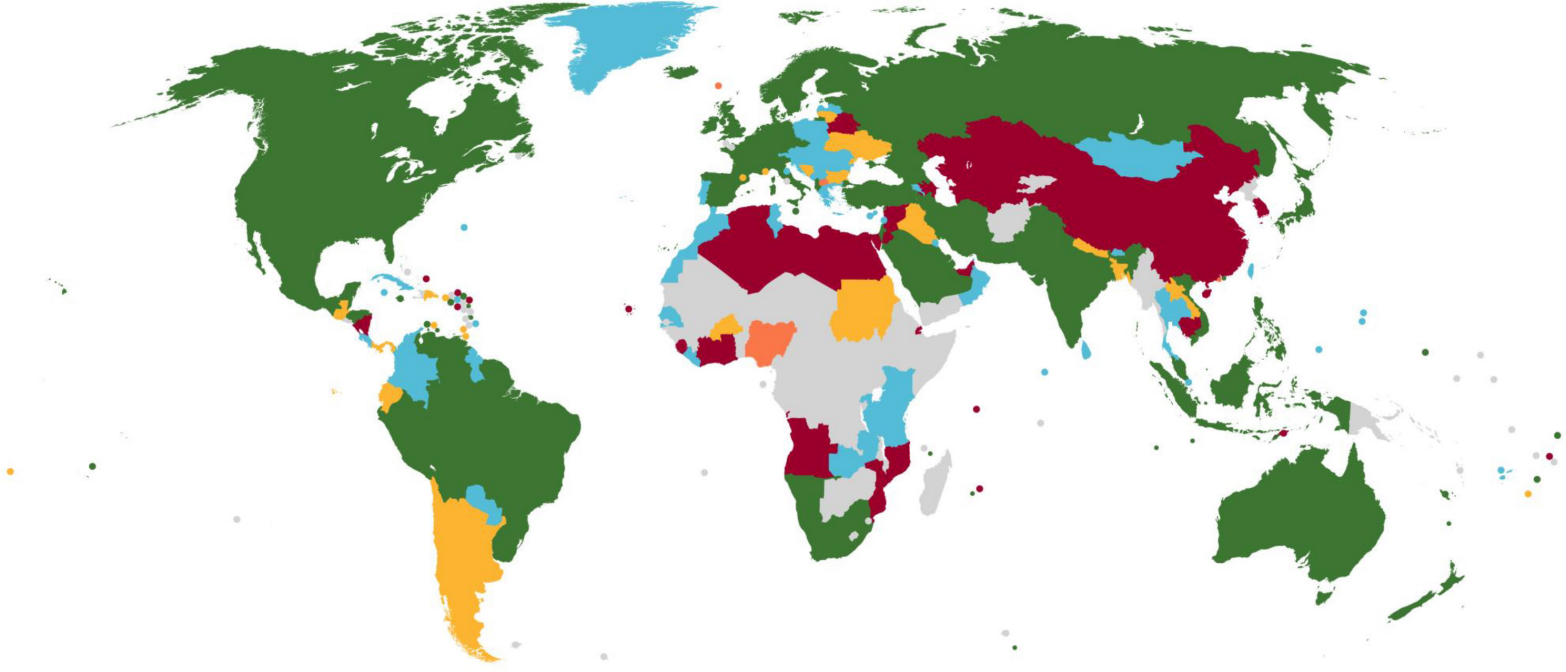
Global snapshot of policies for use of COVID-19 vaccines in pregnancy, 30 September 2021. Map shows the most permissive policy positions for the use of any COVID-19 vaccine in pregnancy for 224 countries and territories, as of 30 September 2021. Colour codes indicate the permissiveness level: green=recommended for some or all; blue=permitted; yellow=permitted with qualifications; orange=not recommended but with exceptions; red=not recommended; grey=no position found.

Figure 2 provides a global snapshot of the most permissive policies in each country/territory and highlights the substantial number of countries for which we could not find pregnancy vaccine policies as of 30 September. Strikingly, only 40% of countries/territories in sub-Saharan Africa had policies for vaccination during pregnancy, compared with 88% in South Asia and 98% in Europe and Central Asia (online supplemental table 1). The majority of high-income countries/territories have policies (85 of 88; 96.6%), whereas the majority of low-income countries do not (8 of 29; 27.6%, p<0.001) (online supplemental table 2). Moreover, the proportion of more permissive policy positions (categories 1 and 2) in LMICs (24.1%) is significantly smaller than in high-income countries (75.0%, p<0.001). The map also highlights heterogeneity in policies within regions. In North America, for example, all countries now have category 1 as their most permissive recommendation, whereas the full range of policy options is represented in East Asia and Pacific. The dynamic evolution of global policies between May and September is captured in 2-week intervals in our online supplemental video 1.

10.1136/bmjgh-2021-007730.supp2 Supplementary data

10.1136/bmjgh-2021-007730.supp1 Supplementary video

### Role of pregnancy-specific data in policy formulation

One hundred seven countries/territories (52%) that issued guidance on COVID-19 vaccine and eligibility referenced the absence of safety or efficacy data in pregnant persons. In some cases, this was cited as the main reason for not recommending vaccination among pregnant persons, as seen in this example from Mozambique in March 2021:

As there are no data on efficacy and safety in specific population groups, children <15 years and pregnant women are excluded from this vaccination process. These groups may be vaccinated later, as soon as scientific evidence of safety and efficacy is produced.^28^

Countries also cited the absence of DART data as a reason to not permit use of certain vaccines in pregnancy. In France (April 2021), for example, guidelines stated:

As a precautionary measure, while awaiting the final results of studies carried out in animals for the AstraZeneca vaccine and in view of the influenza-like syndromes having been reported with this vaccine, it is recommended to give preference to pregnant women mRNA vaccines (Comirnaty or Moderna), or the Covid-19 Janssen vaccine.^29^

Conversely, the increasing availability of observational data on safety in pregnancy collected post-vaccine rollout also appeared to influence countries in the development of more permissive policies. Seventy-four countries/territories (36%) cited observational data, and in some cases, these data were cited as the primary reason for a shift in position, as in the Netherlands in July:

Pfizer and Moderna’s vaccines against COVID-19 are mRNA vaccines. Based on the research data and efficacy of these vaccines, there is no reason to believe that these vaccines would be harmful when used during pregnancy. We now know that 90 000 pregnant women in the United States have been vaccinated with the mRNA vaccines from Pfizer and Moderna. No serious side effects have been reported. That is why it is recommended that all pregnant women - if they are called upon to do so - be vaccinated.^30^

In addition to the observational data regarding COVID-19 vaccine administration in pregnancy, countries also appear to have been influenced by the mounting epidemiological evidence of the risks of SARS-CoV-2 infection in pregnancy: 91 countries/territories (44%) cited these risks in their COVID-19 vaccine policy statements.

### Growing trend for product-specific guidance, with preferential language for some vaccines

Despite the overall increase in the number of policies recommending or permitting use of COVID-19 vaccines during pregnancy, significant differences exist in the number and permissiveness of recommendations by vaccine. Table 2 includes counts of all current policies, both non-specific and product-specific, stratified by level of permissiveness. As of 30 September, policy positions for use in pregnancy have been most frequently articulated for the Oxford/AstraZeneca vaccine, the Pfizer/BioNTech vaccine and the Moderna vaccine (83, 107 and 73 policy positions, respectively; table 2). The frequency of a category 1 recommendation was significantly greater for either the Pfizer/BioNTech vaccine or the Moderna vaccine than for the Oxford/AstraZeneca vaccine (42.1%, 46.6%, 9.6%, respectively, p<0.001); conversely, the frequency of a category 5 recommendation (do not use) was significantly greater for Oxford/AstraZeneca vaccine compared with Pfizer/BioNTech or Moderna (41.0%, 5.6%, 4.1%, respectively, p<0.001). Notably, the frequency of a category 5 recommendation for the Oxford/AstraZeneca vaccine did not change between 21 May (42.9%) and 30 September (41.0%); conversely, the frequency of a category 1 recommendation for Pfizer/BioNTech and Moderna vaccines increased significantly during this same time period (Pfizer: 8.6% to 42.1%, p<0.001; Moderna: 12.5% to 46.6%, p<0.001) (figure 1B–D).

**Table 2:** Variation in permissibility across vaccine product-specific policies, 30 September 2021

Vaccine*	Recommended for some or all		Permitted		Permitted with qualifications		Not recommended but with exceptions		Not recommended		Total
	n	%	n	%	n	%	n	%	n	%	n
Oxford/AstraZeneca	8	9.6	10	12.2	20	24.1	11	13.3	34	41.0	83
Pfizer/ BioNTech	45	442.1	33	30.8	17	15.9	6	5.6	6	5.6	107
Moderna	34	46.6	24	32.9	9	12.3	3	4.1	3	4.1	73
Sinopharm	6	23.1	4	15.4	6	23.1	2	7.7	8	30.8	26
Gamaleya	2	10.0	2	10.0	1	5.0	0	0.0	15	75.0	20
Sinovac	8	44.4	1	5.6	4	22.2	0	0.0	5	27.8	18
Johnson & Johnson	7	18.0	8	20.5	10	25.6	3	7.7	11	28.2	39
Bharat Biotech Covaxin	1	50.0	0	0.0	0	0.0	0	0.0	1	50.0	2
CanSino Biologics Convidecia	2	40.0	1	20.0	1	20.0	0	0.0	1	20.0	5
Non-specific	24	22.4	36	33.6	11	10.3	8	7.5	28	26.2	107

*Includes vaccines where product-specific guidance on the use of the vaccine in pregnancy was available for more than one country/territory.

Some variation in product-specific recommendations may be attributed to the rare thrombotic events (vaccine-induced immune thrombotic thrombocytopenia (VITT)/thrombosis with thrombocytopenia syndrome (TTS)) associated with administration of the Oxford/AstraZeneca and Johnson & Johnson adenovirus-vectored vaccines.^31 32^ In some countries where multiple products are available, the risk of VITT/TTS is cited as a reason to preferentially offer mRNA vaccines to pregnant persons, as seen in this example from Canada (July 2021):

NACI preferentially recommends that a complete vaccine series with an mRNA COVID-19 vaccine should be offered to individuals in the authorized age group who are pregnant or breastfeeding. Informed consent should include discussion about emerging evidence on the safety of mRNA COVID-19 vaccines in these populations… NACI recommends that a viral vector COVID-19 vaccine may be offered to individuals in the authorized age group who are pregnant or breastfeeding to initiate a series when other authorized COVID-19 vaccines are contraindicated or inaccessible. Informed consent should include discussion about the risk and symptoms of VITT, the need to seek immediate medical care should symptoms develop, as well as the limited evidence on the use of viral vector COVID-19 vaccines in these populations.^33^

As of 30 September, 21 countries/territories in our sample had only administered AstraZeneca vaccine^34^; 15 of these (68%) had no available position on vaccine administration in pregnancy. Among countries/territories without mRNA vaccines (72), 50% lacked a pregnancy position, 25% did not recommend administration of any vaccine, and 18% recommended or permitted use of at least one of their available vaccines. Eighty-six per cent of countries/territories with category 4 or 5 positions on use of AstraZeneca in pregnancy had access to mRNA vaccines and 76% of those recommended or permitted use of mRNA vaccines. While countries with available mRNA vaccines chose to give preferential recommendations for their use in pregnancy, other countries without mRNA vaccines, such as India, Iran, Venezuela and Zambia, also shifted to more permissive policies (figure 1B).

## Discussion

Increasing evidence of the safety of COVID-19 vaccines in pregnancy supports the conclusion that the benefits outweigh the risks whenever there is ongoing or anticipated community transmission. We documented and categorised pregnancy-specific COVID-19 vaccination policies issued by PHAs in 176 countries and by the WHO. We found marked changes in policies over time. Recommendations became substantially more permissive, with 32% permitting or recommending at least one COVID-19 vaccine in May, and 66% doing so as of 30 September. Among countries issuing pregnancy-specific policies, one-third noted the availability of observational safety data and almost half cited evidence of the risks of SARS-CoV-2 infection in pregnancy in their rationale for recommending or permitting use. Nevertheless, even at the end of the study period, 17% of national policies continued to recommend against the use of any COVID-19 vaccine during pregnancy. Importantly, gaps in policies appeared to exist regionally and in poorer countries, with policies available in only 40% of sub-Saharan African countries and 28% of low-income countries. These disparities are likely explained, at least in part, by delays in country-wide vaccine access but may also be related to an absence of pregnancy-specific data for some of the vaccines available in these countries. Further work is needed to delineate the range of reasons for these policy gaps.

Our findings raise concern about equitable access to COVID-19 vaccines during pregnancy. In many countries, pregnant people are less likely than other groups who are also at increased risk of serious disease, and even those groups not at increased risk, to have access to COVID-19 vaccines.

Globally, inequities also exist *among* pregnant people, as well as *between* pregnant people and other groups. As of 30 September, the Oxford-AstraZeneca vaccine was the most commonly administered COVID-19 vaccine in the world (172 countries),^34^ but far fewer countries/territories explicitly recommended or permitted its use in pregnant people without qualifications compared with the Moderna or Pfizer vaccines. Pregnant people living in largely low-income parts of the world, where mRNA vaccines are in limited supply, were less likely than pregnant people living in high-income countries, where mRNA vaccines are widely available, to have access to COVID-19 vaccines. Thus, the global inequity in access to vaccines in pregnancy does not merely reflect global injustices in vaccine supply, it is an inequity that is further compounded by disparities between higher-income and lower-income countries in the types of vaccines that are locally available.

Previously, we and others developed the Pregnancy Research Ethics for Vaccines, Epidemics, and New Technologies (PREVENT) Guidance for inclusion of the interests of pregnant individuals in development and deployment of vaccines against emerging pathogens.^5^ In the PREVENT Guidance, which was developed prior to the COVID-19 pandemic, we noted that standard approaches to determining whether pregnant people could be offered vaccines in the research context typically operated on a presumption of exclusion, in which the default position was to deny access. We described a cascade in which the presumption of exclusion of pregnant people from vaccine trials resulted in the absence of data specific to pregnancy, leading to exclusion of pregnant people from vaccine deployment activities. Unfortunately, the uniform exclusion of pregnant people from COVID-19 vaccine trials prior to authorisation has led to a predictable scenario in many countries, in which countries clearly articulate the absence of clinical trial data in their rationale for restricting access to COVID-19 vaccines for pregnant people. Another specific PREVENT recommendation, that DART studies be completed as early as possible and preferably before the onset of phase 3 trials, was also not heeded. The fact that no DART data were available for several COVID-19 vaccines until months after emergency use authorisation/listing was granted was noted by multiple countries as a reason for restrictive pregnancy policies. The subsequent availability of observational data for some vaccines has led some countries to modify their positions in favour of broadly permitting or recommending vaccination; however, the scarcity of data for many vaccines, and the absence of evidence from LMIC settings, have meant that the most permissive recommendations focus principally on Pfizer or Moderna vaccines, which, as of this writing, are available in only a few LMICs.^34^

Our study has a number of limitations. First, country policies regarding the use of COVID-19 vaccines are continuously evolving. Our data only provide a snapshot of dynamic global policymaking over a short time period. To continue tracking global variance in public health guidelines for COVID-19 vaccine use in pregnancy, we have developed an online tracker (www.comitglobal.org), in which data are updated at least every 3 weeks. Second, we were unable to find policies on COVID-19 vaccines and pregnancy for 48 countries/territories, many of them in Africa. This may initially have been related to limited vaccine access; however, most countries have introduced at least one COVID-19 vaccine but policies on administration in pregnancy continue to lag. It is also possible that countries with fewer resources may not regularly post their policies or recommendations on their online platforms, so that we may not have captured policies that were developed and disseminated through other channels. As we identify updates for these and other countries, they will be posted to the COMIT tracker (www.comitglobal.org).

Although the WHO Prioritization Roadmap recommends that pregnant people receive COVID-19 vaccines at the same time as other people who are at elevated risk of severe disease and death,^26^ 17% of countries still recommended against any use of COVID-19 vaccines in pregnancy as of late September 2021. In many instances, these recommendations were product or platform specific and referred to adenovirus-vectored vaccines. The categorical exclusion of pregnant people from adenovirus-vectored vaccine rollout in settings with community transmission and where no alternative vaccines are available is ethically unjustifiable.^5^ Of note, the WHO specifically rejects this position. In its interim recommendations for both the Oxford/AstraZeneca and the Janssen/J&J vaccine,^35 36^ the WHO allows for the administration of these vaccines in pregnancy when the benefits of vaccination outweigh the risks, as they likely do in many high-transmission settings with no or insufficient vaccine alternatives. Because the epidemiology of the pandemic can change rapidly, as has recently occurred with the Omicron variant, it is our position that pregnant people should have access to vaccines wherever there is ongoing or anticipated community transmission.

In some countries, vaccines are in such short supply that pregnant people are only one among many higher-risk groups who have no access. But constrained national supply is only part of the story. In some countries, pregnant people are being denied access even when they are members of high priority groups, like health workers, who are being offered vaccines. In still other countries, immunisation programmes are offering vaccines to groups at elevated risk of severe disease and death, but not to pregnant people who, as a group, also fit this description. As evidence continues to mount regarding the harms of COVID-19 in pregnancy, including increasing rates of maternal mortality,^27 37^ national policymakers must include pregnant people in their prioritisation plans and work to increase demand and uptake in this group.

## Conclusion

Despite improvements in country policies for inclusion of pregnant people in COVID-19 vaccine deployment, substantial gaps remain in low-income countries and in countries in sub-Saharan Africa. To address and resolve these inequities for future outbreaks and pandemics, it is imperative that DART data and data on vaccine safety in pregnancy be collected early enough in the vaccine research and development process to provide country policymakers, clinicians, and pregnant people the evidence they need to make sound and fair decisions about use of vaccines in pregnancy. As we have noted previously, the presumption of inclusion of pregnant people in clinical vaccine trials and in vaccine deployment is critical for ensuring equitable access in these circumstances,^5^ and upholds the principles of WHO Immunization Action 2030, A Global Strategy to Leave No One Behind.^38^

## Data Availability

Data are available upon reasonable request. Our data collection protocol and data used in these analyses are available at comitglobal.org.
