# Supplementary material for: Global disparities in public health guidance for the use of COVID-19 vaccines in pregnancy
Source: BMJ Glob Health. 2022 Feb 24;7(2):e007730. doi: 10.1136/bmjgh-2021-007730 (PMC8882664; doi:10.1136/bmjgh-2021-007730)
Supplement: Supplementary data [file bmjgh-2021-007730supp002.pdf]

Supplementary Material: Global Disparities in Public Health Guidance for the use of COVID-19 Vaccines in Pregnancy

Table of Contents

Supplementary Table 1. Country policies for use of COVID-19 vaccines in pregnancy by region, September 30th, 2021 2

Supplementary Table 2. Country policies for use of COVID-19 vaccines in pregnancy by income-level, September 30th, 2021 3

Zavala E, *et al. BMJ Global Health* 2022; 7:e007730. doi: 10.1136/bmjgh-2021-007730

Supplementary Table 1. Country policies for use of COVID-19 vaccines in pregnancy by region, September 30th, 2021

	Country/territory counts of policy positions, n (%)							
Region	1-Recommended	2-Permitted	3-Permitted with qualifications	4-Not recommended but with exceptions	5-Not recommended	6-No position found	0-No data	Total
Sub-Saharan Africa	2 (4.2)	6 (12.5)	3 (6.3)	1 (2.1)	7 (14.6)	16 (33.3)	13 (27.1)	48
South Asia	2 (25.0)	3 (37.5)	2 (25.0)	0 (0.0)	0 (0.0)	1 (12.5)	0 (0.0)	8
Latin America and Caribbean	15 (33.3)	9 (20.0)	11 (24.4)	0 (0.0)	4 (8.9)	4 (8.9)	2 (4.4)	45
East Asia and Pacific	14 (34.1)	8 (19.5)	4 (9.8)	1 (2.4)	5 (12.2)	7 (17.1)	2 (4.9)	41
Middle East and North Africa	6 (28.6)	5 (23.8)	1 (4.8)	0 (0.0)	7 (33.3)	2 (9.5)	0 (0.0)	21
Europe and Central Asia	24 (41.4)	19 (32.8)	6 (10.3)	2 (3.4)	6 (10.3)	1 (1.7)	0 (0.0)	58
North America	2 (66.7)	1 (33.3)	0 (0.0)	0 (0.0)	0 (0.0)	0 (0.0)	0 (0.0)	3
Total	65 (29.0)	51 (22.8)	26 (11.6)	4 (1.8)	29 (12.9)	31 (13.8)	18 (8.0)	224

Zavala E, *et al. BMJ Global Health* 2022; 7:e007730. doi: 10.1136/bmjgh-2021-007730

Supplementary Table 2. Country policies for use of COVID-19 vaccines in pregnancy by income-level, September 30th, 2021

	**Country/territory counts of policy positions, n (%)**							
Country/ territory Income Level	1: Recommended	2: Permitted	3: Permitted with qualifications	4: Not recommended but with exceptions	5: Not recommended	6: No position found	0: No data	Total
Low	0 (0.0)	2 (6.9)	2 (6.9)	0 (0.0)	4 (13.8)	12 (41.4)	9 (31.0)	29
Lower Middle	7 (14.0)	10 (20.0)	5 (10.0)	1 (2.0)	11 (22.0)	11 (22.0)	5 (10.0)	50
Upper Middle	19 (33.3)	12 (21.1)	9 (15.8)	1 (1.8)	8 (14.0)	5 (8.8)	3 (5.3)	57
High	39 (44.3)	27 (30.7)	11 (12.5)	2 (2.3)	6 (6.8)	3 (3.4)	0 (0.0)	88
Total	65 (29.0)	51 (22.8)	26 (11.6)	4 (1.8)	29 (12.9)	31 (13.8)	18 (8.0)	224

Zavala E, *et al. BMJ Global Health* 2022; 7:e007730. doi: 10.1136/bmjgh-2021-007730
